# IV期肺腺癌伴神经内分泌分化出现PCT异常升高1例并文献复习

**DOI:** 10.3779/j.issn.1009-3419.2026.106.17

**Published:** 2026-06-20

**Authors:** Yongquan PAN, Ting WU, Jinfeng JIANG, Tianyu SUN, Chenyang LIU, Hao DING

**Affiliations:** ^1^212002 镇江，江苏大学附属人民医院呼吸与危重症医学科（潘勇全，蒋金凤，孙天宇，刘晨阳，丁浩）; ^1^Department of Respiratory and Critical Care Medicine; ^2^病理科（吴婷）; ^2^Department of Pathology, Affiliated People’s Hospital of Jiangsu University, Zhenjiang 212002, China

**Keywords:** 肺肿瘤, 降钙素原, 神经内分泌分化, 预后, 疗效监测, Lung neoplasms, Procalcitonin, Neuroendocrine differentiation, Prognosis, Treatment response monitoring

## Abstract

降钙素原（procalcitonin, PCT）是临床用于细菌感染诊断、抗菌疗效评估及预后判断的关键生物标志物。研究发现，PCT在多种恶性肿瘤中亦可升高，与肿瘤进展、转移及患者不良预后密切关联。然而，PCT对于非小细胞肺癌（non-small cell lung cancer, NSCLC）病情及疗效监测的价值尚未明确。本文报道1例经治IV期肺腺癌伴神经内分泌分化患者，在无活动性感染证据的情况下出现血清PCT异常升高，对胸膜转移灶行局部放疗后PCT水平迅速下降。本文旨在探讨PCT在NSCLC伴神经内分泌分化中的潜在临床意义，为类似患者的病情评估及预后判断提供参考依据。

## Abstract (en)

Procalcitonin (PCT) is a key biomarker widely used in clinical practice for the diagnosis of bacterial infection, assessment of antimicrobial efficacy, and prognostic evaluation. Studies have shown that PCT can also be elevated in various malignancies, correlating closely with tumor progression, metastasis, and poor prognosis. However, the value of PCT in monitoring disease status and treatment response in non-small cell lung cancer (NSCLC) remains unclear. This article reports a case of a patient with previously treated stage IV lung adenocarcinoma with neuroendocrine differentiation, who presented with a marked elevation in serum PCT in the absence of active infection. Following local radiotherapy to the pleural metastatic lesion, the PCT level declined rapidly. This report aims to explore the potential clinical significance of PCT in NSCLC with neuroendocrine differentiation and to provide a reference for disease assessment and prognostic evaluation in patients with similar symptoms.

降钙素原（procalcitonin, PCT）是一种由116个氨基酸组成的蛋白质，作为降钙素的前体物质，其主要由甲状腺C细胞合成，在健康人群血液中水平极低，通常<0.1 ng/mL^[1]^。在细菌感染状态下，其水平可急剧升高，使其成为诊断脓毒症、评估感染严重程度及指导抗生素治疗的重要生物标志物^[1]^。然而，近年来研究^[2]^发现，PCT升高不仅限于感染状态，在无感染证据的恶性肿瘤患者中亦可出现，尤其在肿瘤负荷较大或存在远处转移时更为显著，这与多种恶性肿瘤的进展、转移及不良预后密切关联。

肺癌是全球发病率和死亡率最高的恶性肿瘤^[3]^，其病理类型主要包括非小细胞肺癌（non-small cell lung cancer, NSCLC）和小细胞肺癌（small cell lung cancer, SCLC）。其中，SCLC因具有神经内分泌特性，PCT常显著升高，且已被证实为独立的负面预后因素^[4]^。相比之下，有研究^[5]^发现PCT升高亦与NSCLC预后不良有关，但其是否可作为NSCLC的病情与疗效监测指标，目前尚不清楚。本文报道1例IV期肺腺癌伴神经内分泌分化患者，在无明显感染证据下PCT持续异常升高，而在明确胸膜转移并行局部放疗后PCT水平迅速下降。结合文献复习，本文旨在探讨PCT在NSCLC中异常升高的潜在原因，并分析其作为病情监测、疗效评估及预后判断指标的临床价值。

## 1 病例资料

患者男，69岁，无吸烟史，2017年8月10日因体检查胸部计算机断层扫描（computed tomography, CT）示“右肺中叶占位”至江苏省肿瘤医院行肺穿刺活检，诊断为右肺腺癌（cT4N2M0，IIIB期），下一代测序（next-generation sequencing, NGS）示驱动基因阴性。随后就诊于江苏大学附属人民医院，2017年8月25日起行“培美曲塞0.8 g d1+顺铂35 mg d1-4 +贝伐珠单抗400 mg d1, *q3w*”治疗4个周期（图1），疗效评估为部分缓解（partial response, PR）。2017年12月6日起规律行“培美曲塞0.8 g d1+贝伐珠单抗400 mg d1, *q4w*”维持治疗（图1），疗效评估为疾病稳定（stable disease, SD）。2021年6月9日患者胸部CT示右侧胸腔积液伴局部胸膜增厚（图2A），胸水脱落细胞倾向腺癌，胸水NGS示棘皮动物微管相关蛋白样4-间变性淋巴瘤激酶（echinoderm microtubule associated protein like 4-anaplastic lymphoma kinase, *EML4-ALK*）（exon6-exon20）融合突变阳性（图1），遂修正诊断为右肺腺癌（cT4N2M1a，胸膜，IVA期），先后口服阿来替尼、布格替尼、伊鲁阿克靶向治疗。2025年5月12日患者因“咳嗽咳痰”就诊于江苏大学附属人民医院，查PCT水平明显升高（34.13 ng/mL）（图1），但患者无发热、寒战、胸痛、腹痛腹泻、尿频尿急尿痛、皮肤红肿等感染相关症状，C反应蛋白、白细胞计数、中性粒细胞计数、白细胞介素-6（interleukin-6, IL-6）、肿瘤坏死因子-α（tumor necrosis factor-α, TNF-α）、白细胞介素-1β（interleukin-1β, IL-1β）等炎症相关指标正常，痰涂片、痰培养及呼吸道病原体抗体谱均阴性，予以经验性抗感染治疗，患者咳嗽咳痰好转。2025年6月12日复查PCT升高至42.83 ng/mL（图1），胸部CT示右侧胸腔包裹性积液伴胸膜增厚（图2B）。为进一步评估病情，2025年6月26日完善正电子发射计算机断层显像（positron emission tomography/CT, PET/CT）示右侧胸膜腔内壁层胸膜下不规则软组织密度肿块及结节样胸膜增厚，氟代脱氧葡萄糖（fluorodeoxyglucose, FDG）代谢增高，考虑为胸膜转移（图3B），较2024年9月24日PET/CT（图3A）明显进展。遂行超声引导下胸膜穿刺活检，病理示（右侧胸膜）低分化癌，倾向非小细胞癌（图4A、4B）。免疫组化示：突触素（synaptophysin, Syn）、癌胚抗原（carcinoembryonic antigen, CEA）、广谱细胞角蛋白（cytokeratin pan, CKpan）、细胞角蛋白7（cytokeratin 7, CK7）、CK18、甲状腺转录因子-1（thyroid transcription factor-1, TTF-1）、核增殖抗原（Ki-67 proliferation index, Ki-67）40%、嗜铬粒蛋白A（chromogranin A, CgA）、CD56、胰岛素瘤相关蛋白1（insulinoma-associated protein 1, INSM1）阳性（图4C-4F），结合病史考虑肺腺癌转移，伴神经内分泌分化。NGS示*ALK *exon23 c.3604G>A, p.G1202R突变、*EML4-ALK*（exon6-exon20）融合突变阳性（图1）。考虑患者胸膜病灶寡进展，于2025年7月29日起行右侧胸膜转移灶放疗，剂量60 Gy/30 Fx（图1）。2025年10月9日复查PCT水平降至6.07 ng/mL（图1），胸部CT较2025年6月12日局部胸膜增厚改善，胸腔积液减少（图2C）。后续患者继续口服伊鲁阿克靶向治疗至今，评估疗效为SD。本病例报道已获得患者知情同意，相关内容通过江苏大学附属人民医院伦理委员会审批，伦理审批号为[2026]KYW001-59。

**图 1:**
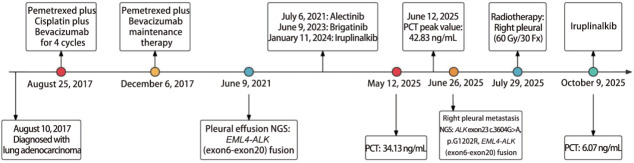
患者治疗时间线

**图 2:**
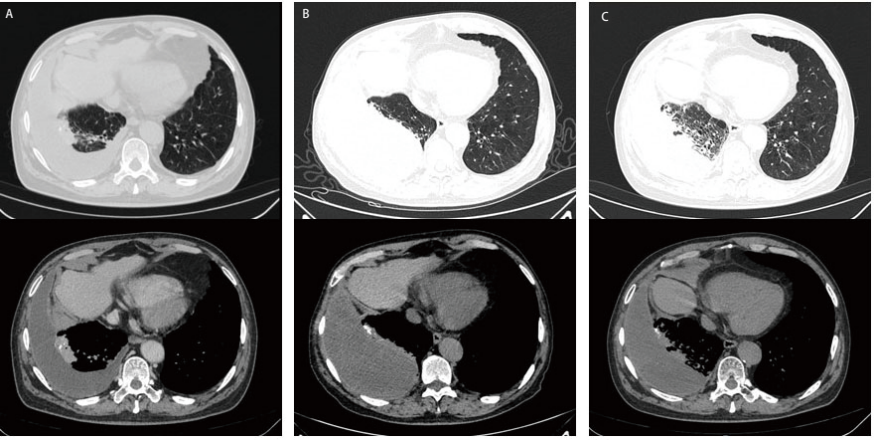
患者治疗期间的胸部CT图片。A：2021-06-09：右侧胸腔积液伴局部胸膜增厚；B：2025-06-12：右侧胸腔包裹性积液伴胸膜增厚；C：2025-10-09：胸膜病灶放疗后局部胸膜增厚改善，胸腔积液减少。

**图 3:**
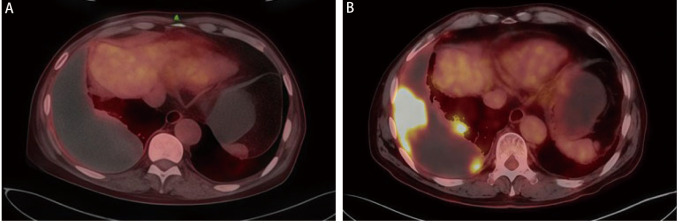
患者治疗期间的PET/CT图片。A：2024-09-24：右侧局部胸膜增厚，FDG代谢增高，考虑胸膜转移可能，右侧胸腔可见包裹性积液，内部密度不均，FDG代谢未见明显增高；B：2025-06-26：右侧胸膜腔内壁层胸膜下不规则软组织密度肿块及结节样胸膜增厚，FDG代谢增高，考虑胸膜转移，较之前（2024-09-24）明显进展。

**图 4:**
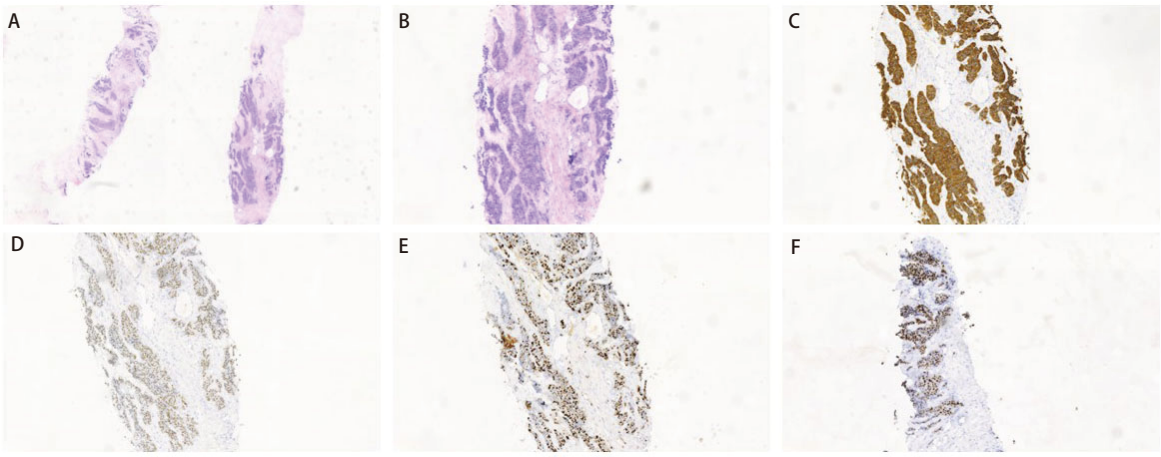
右侧胸膜转移灶的组织病理学和免疫组织化学特征。A，B：（右侧胸膜）转移性肺腺癌（HE染色，A：×40，B：×100）；C：Syn阳性（IHC染色，×100）；D：TTF-1阳性（IHC染色，×100）；E：Ki-67 40%阳性（IHC染色，×100）；F：INSM1阳性（IHC染色，×100）。

## 2 讨论

肺癌是全球发病率和死亡率最高的恶性肿瘤^[3]^，主要分为NSCLC和SCLC。SCLC因具有神经内分泌特性可引起PCT升高。早在1986年，Cate等^[6]^即发现SCLC细胞系可合成并释放PCT。Patout等^[7]^对147例肺癌患者的回顾性分析显示，在排除感染因素后SCLC及含有神经内分泌成分的大细胞癌患者PCT水平显著高于其他类型肺癌患者。既往多项研究^[4,8]^证实PCT升高与SCLC的预后不良显著关联。相比之下，NSCLC中PCT升高较为少见，Ermayanti等^[9]^报道1例肺鳞癌伴骨、脑转移患者，PCT水平>200 ng/mL，因病情迅速进展而死亡。Kajikawa等^[5]^发现NSCLC患者PCT升高与感染无关，且PCT升高组患者体能状态更差，总生存期更短，提示PCT升高同样可作为NSCLC预后不良的指标。

然而，PCT是否可作为NSCLC的病情与疗效监测指标目前尚不清楚。SCLC患者在接受有效的抗肿瘤治疗后PCT水平显著下降。Pardo-Cabello等^[10]^报道1例广泛期SCLC伴脑、骨、纵隔转移患者，在排除感染后PCT仍进行性升高（最高达2.14 ng/mL），而在接受化疗和全脑放疗后肿瘤较前缩小且PCT恢复正常。本例患者PCT在短期内迅速升高，与影像学提示疾病进展基本一致，而在针对胸膜转移灶行局部放疗后PCT水平明显下降，胸部CT亦提示胸膜转移及胸腔积液有所控制，提示PCT下降可能与抗肿瘤疗效有关。因此，本课题组认为PCT或可作为NSCLC（尤其是伴有神经内分泌分化的NSCLC）患者病情与疗效的监测指标。Chen等^[11]^在消化系统神经内分泌肿瘤中发现血清PCT水平的变化与治疗反应有关，PR和SD患者的血清PCT水平显著下降，而当患者出现疾病进展时，血清PCT会再次显著升高。不过，目前关于血清PCT水平与伴有神经内分泌分化的NSCLC治疗反应间关联的研究数据仍十分缺乏，未来需进一步探索。

关于NSCLC患者PCT升高的具体机制尚不完全清楚。有研究^[12]^认为与肿瘤微环境中的炎症状态、缺氧等因素有关，这些因素可诱导炎症因子（如IL-6、TNF-α、IL-1β等）的产生，进而刺激PCT升高。然而，本例患者上述炎症因子均未见升高，提示其PCT升高可能主要源于肿瘤自身。PCT可由甲状腺C细胞以及分布于肺、肾上腺、肝脏、肾脏和肌肉中的神经内分泌细胞分泌^[13]^，该患者胸膜转移灶活检提示其具有神经内分泌分化特征，故PCT升高可能与神经内分泌密切相关。这与先前的研究^[7]^一致，即在含有神经内分泌成分或伴有远处转移的肺癌中PCT显著升高。另外，Mohamed等^[14]^比较了肺癌合并特发性肺纤维化（lung cancer combined with idiopathic pulmonary fibrosis, LC-IPF）与单纯IPF患者的血清PCT水平，发现LC-IPF患者的血清PCT水平显著高于单纯IPF患者，推测PCT升高可能与神经内分泌成分、癌症晚期及多发性转移有关。本例患者为IV期NSCLC，在胸膜病灶明显进展的同时，出现PCT水平异常升高，而在放疗后显著下降。因此，本文作者推测该患者PCT升高为神经内分泌分化、胸膜转移及癌症晚期共同作用所致。

值得注意的是，本例患者病程长，历经化疗及多种靶向药物治疗，需考虑是否存在NSCLC向SCLC转化的可能。组织学转化是NSCLC在接受靶向治疗后获得性耐药的重要机制之一，尤其在表皮生长因子受体（epidermal growth factor receptor, *EGFR*）突变阳性肺腺癌中报道较多，但*ALK*融合阳性患者中亦有发生。转化发生的分子基础通常涉及视网膜母细胞瘤基因1（retinoblastoma 1,* RB1*）和肿瘤蛋白53（tumor protein 53, *TP53*）的共同失活，此外磷酸酶及张力蛋白同源物（phosphatase and tensin homolog, *PTEN*）、Notch受体1（Notch receptor 1, *NOTCH1*）失活及*MYC*通路激活也可能参与其中^[15]^。然而，本例患者先后3次NGS检测均未检出*EGFR*、*RB1*、*TP53*、*PTEN*、*NOTCH1*等突变，且胸膜转移灶穿刺病理仍为腺癌，未呈现典型SCLC形态，组织学转化的分子与病理依据均不充分。此外，神经元特异性烯醇化酶（neuron-specific enolase, NSE）及胃泌素释放肽前体（pro-gastrin-releasing peptide, pro-GRP）是监测SCLC转化的有效血清学指标，研究^[15]^发现其在转化后常显著升高，而本例患者该两项指标始终在正常范围内，进一步支持组织学转化可能性较低。不过，本例患者免疫组化显示Syn、CgA、CD56、INSM1等神经内分泌标志物阳性，提示肿瘤具有神经内分泌分化潜能，但PCT能否作为早期预测NSCLC向SCLC转化的血清学指标，尚需深入研究。鉴于PCT检测简便、经济、可动态监测，本文作者认为，在伴有神经内分泌分化特征的NSCLC患者中，若出现PCT进行性升高而缺乏感染证据时，应警惕肿瘤负荷增加乃至组织学转化的可能，建议及时完善影像学，必要时再活检以明确病情。

综上，本例IV期肺腺癌患者在无明显感染证据的情况下出现PCT显著升高，考虑与肿瘤神经内分泌分化特性及肿瘤负荷增加有关。此外，PCT的动态变化可能具有一定的病情监测和疗效评估价值。因此，对于伴有神经内分泌分化特征的晚期肺癌患者，PCT水平及其动态变化可能具有评估肿瘤负荷与治疗反应的潜在临床价值，但仍需大规模前瞻性研究评估PCT作为此类患者疗效评估和预后预测生物标志物的敏感性与特异性。
